# Piloting a New Model for Treating Music Performance Anxiety: Training a Singing Teacher to Use Acceptance and Commitment Coaching With a Student

**DOI:** 10.3389/fpsyg.2020.00882

**Published:** 2020-05-28

**Authors:** Teresa A. Shaw, David G. Juncos, Debbie Winter

**Affiliations:** ^1^Department of Music, University of Chichester, Chichester, United Kingdom; ^2^Centre for Voice Studies, East Bergholt, United Kingdom; ^3^Hornstein, Platt & Associates, Counseling and Wellness Centers, Philadelphia, PA, United States; ^4^Wales Academy for Professional Practice and Applied Research, University of Wales Trinity Saint David, Carmarthen, United Kingdom

**Keywords:** Acceptance and Commitment Therapy, Acceptance and Commitment Coaching, vocal student, psychological flexibility, music performance anxiety

## Abstract

Thus far, treatments for music performance anxiety (MPA) have focused primarily on interventions administered by psychologists and mental health clinicians with training and education in psychotherapy. While these interventions are promising or even efficacious, many musicians prefer not to work with a psychotherapist due to stigma and lack of time/access. Student musicians are particularly vulnerable to developing MPA, and while they may prefer consulting with their teachers about MPA over psychotherapists, many teachers feel unqualified to help. Here, we investigated an alternative intervention model, in which a clinical psychologist with MPA expertise trained a singing teacher with no training or education in psychotherapy to use an evidence-based coaching model, Acceptance and Commitment Coaching (ACC), with a student vocalist with problematic MPA, in a single-subject design format. ACC is a version of Acceptance and Commitment Therapy (ACT) that has been used under various names with non-clinical populations to help enhance psychological flexibility, e.g., with athletes, at the workplace, with undergraduates, and others. The teacher received approximately seven hours of ACC training via Skype. In turn, she provided six one-hour ACC sessions to a university student vocalist. Materials for the training and coaching sessions were taken from an ACC book and an ACT-based self-help book for musicians, and the teacher also adhered to a GROW model of coaching. The student made clinically significant improvements in two ACT-based processes believed to correlate with improved psychological flexibility in previous ACT for MPA psychotherapy research, i.e., acceptance of MPA-related discomfort and defusion from MPA-related thoughts. The student also reported a significant shift had occurred in his thinking: he became more willing to have his MPA, and so he volunteered to sing in classes early in the upcoming semester, and he auditioned for & won a lead role in a musical, both of which he previously avoided doing. ACC appears to be a promising MPA intervention that can be administered by a music teacher without training or education in psychotherapy, and it may help schools who do not employ psychologists and are therefore unable to follow best practice guidelines for treating MPA.

## Introduction

### MPA and Available Treatments

Music performance anxiety (MPA) is a common type of anxiety that affects musicians of all skill levels and can shorten music careers if not properly treated. Prevalence estimates for MPA in its more problematic form are approximately 15–25% for professional musicians (Fishbein et al., 1988) and 20–35% for university musicians (Wesner et al., 1990; Schroeder and Liebelt, 1999; Kaspersen and Gotestam, 2002). Compared to professionals, university-aged musicians may experience a slightly higher prevalence of problematic MPA, because musicians under age 30 are known to be at increased risk for experiencing MPA (Kenny et al., 2012), and university students often have less experience performing music at high levels (Papageorgi et al., 2011; Biasutti and Concina, 2014). In distinguishing between more and less problematic forms of MPA, some researchers (Juncos and de Paiva e Pona, 2018) recommend looking for the presence of four symptom categories to aid in this assessment, that is, *cognitive symptoms* such as worry about making a mistake and the implications of making a mistake and narrowing of one’s attention onto actual or perceived threats in the performance; *physiological arousal symptoms* such as shortness of breath, palpitations, tachycardia, tightness in the chest, and dry mouth; *behavioral avoidance and/or anxious behaviors* such as overtly avoiding solos and auditions or avoiding performing entirely and/or more covertly avoiding challenging oneself with new repertoire, avoiding making eye contact with jurors or audience members, and avoiding expressing oneself more. When performing is unavoidable, the musician usually displays *anxious behaviors* in addition to covert behavioral avoidance. Common examples of anxious behaviors include physical manifestations of anxiety (fidgeting, repetitive hand or body movements, wringing hands, shaky hands or feet, pulling on earlobe, etc.), verbal manifestations (talking faster, talking more loudly or forcefully, stammering, stuttering, inflecting pitch upward, rambling, pausing or hesitating to speak, etc.), and facial manifestations (tensing eye muscles, blinking often, wincing, opening eyes widely, biting lips, tilting head back or pulling head forward on top of the neck, etc.). The fourth category to look for is *distress and/or impairment associated with having MPA*, such as feeling frustrated, depressed, or ashamed for having MPA and/or enduring new problems due to MPA, for example, needing to add another semester to one’s education due to avoiding too many required performances. The more categories of MPA symptoms one experiences, the more problematic the MPA. Lastly, MPA usually occurs in performance settings in which there is high ego involvement, an evaluative threat from an audience, and a fear of failure (Kenny, 2011).

Thus far, treatments for MPA have focused primarily on using psychotherapeutic and medicinal interventions, both of which must be administered by qualified practitioners with proper education and training in clinical psychology, medicine, or other health disciplines. There are several psychotherapeutic options to consider for treating MPA, and cognitive behavioral therapy (CBT) is considered best practice by leading MPA researchers (Kenny, 2011), particularly when it includes exposure therapy, a common form of behavioral therapy in which one is continually exposed to a feared stimulus(i) until their anxiety habituates. Currently, CBT with exposure has the strongest research support as an MPA treatment (Kenny, 2011). For a review of the studies using CBT to treat MPA, see Kenny (2011). Other forms of psychotherapy that have been studied as MPA treatments are acceptance and commitment therapy (ACT) (Erenius and Wallengren, 2012; Juncos et al., 2014, 2017; Juncos and Markman, 2015; Clarke et al., 2019) and psychodynamically oriented therapy (Kenny et al., 2014, 2016; Kenny, 2016). ACT shows promise in treating problematic cases of MPA (Erenius and Wallengren, 2012; Juncos et al., 2014, 2017; Juncos and Markman, 2015; Clarke et al., 2019), whereas intensive short-term dynamic psychotherapy (ISTDP) shows promise in treating more severe forms of MPA with accompanying panic and depression (Kenny et al., 2014, 2016; Kenny, 2016). By far, the most common medicinal treatment for MPA is beta-adrenoceptor blocking medication, or “beta-blockers.” As many as 31% of professional, orchestral musicians use beta-blockers to reduce the physiological arousal symptoms associated with MPA, for example, propranolol and nadolol (Kenny et al., 2012). Unfortunately, they are contraindicated for vocalists, woodwind and brass instrumentalists, and musicians with breathing disorders like asthma due to their constriction of the airways (Kenny, 2011), leaving out numerous musicians who might prefer them due to their widespread acceptance among professional musicians.

Although the aforementioned interventions are promising or even efficacious in treating problematic MPA, that is, CBT with exposure, many musicians prefer not to work with a mental health clinician or a physician to treat their MPA due to stigma or to a lack of time/access to services. Certainly, alternative MPA treatments may lessen the stigma of psychotherapy, such as biofeedback (Egner and Gruzelier, 2003; Thurber et al., 2010), yoga (Khalsa et al., 2009), meditation (Lin et al., 2008), the Alexander Technique (Valentine et al., 1995; Egner and Gruzelier, 2003; Hoberg, 2008), and guided imagery with progressive muscle relaxation (Sisterhen, 2005; Kim, 2008) or guided imagery alone (Esplen and Hodnett, 1999). However, the research support for these interventions is not as strong as that for psychotherapy or beta-blockers (Juncos and de Paiva e Pona, 2018) and these also require a similar time commitment and access to qualified practitioners.

### An Alternative Model for MPA Treatment

While research for available MPA treatments has grown considerably over the past 20 years, one area that has received little attention is how music teachers may administer interventions themselves. A review of the studies cited herein revealed that few MPA interventions were administered by music teachers, with the exceptions of the Alexander Technique (Hoberg, 2008) and guided imagery with progressive muscle relaxation (Sisterhen, 2005). The overwhelming majority of interventions were administered by psychologists or other healthcare practitioners. This is unfortunate, given how teachers have regular access to student musicians who need help due to being at higher risk for problematic MPA and that students may even *prefer* to consult with their teachers instead of healthcare practitioners (Williamon and Thompson, 2006). Simultaneously, many teachers feel a strong need to help, but they believe they are unqualified due to their lack of education and training in psychotherapy or other health disciplines. While this belief is understandable, it is not fully accurate. Certainly, teachers can receive training in alternative treatments like the Alexander Technique (Hoberg, 2008) and guided imagery with progressive muscle relaxation (Sisterhen, 2005). However, in order to properly administer these interventions, one needs adequate training and a certification in the case of the Alexander Technique, both of which are time-consuming for teachers.

Here, we propose a different model for MPA intervention that involves music teachers but may be less time-consuming: training them in an evidence-based coaching model. In adapting interventions used with athletes for use with musicians, sport and performance psychologists have started to train music teachers in coaching interventions already shown to be effective for enhancing athletic performance (Daubney and Daubney, 2017). We agree with this approach and encourage teachers to receive training in sport and performance coaching interventions, whenever possible, because we believe the aforementioned hurdles that prevent musicians from seeking help may be lessened when students work with their teachers. However, training music teachers in a specific, evidence-based coaching model may be more beneficial than providing them with broad, performance enhancement training that pulls from many models, because they may feel overwhelmed by the need to learn information from multiple models.

### Acceptance and Commitment Coaching as a Treatment for MPA

One specific coaching model that has shown promise in treating MPA and enhancing performance is ACT. Like CBT, ACT (pronounced as the word “act”) is a behavioral therapy that uses exposure and other behavioral principles to evoke positive change. However, ACT is different than CBT because it does not aim to control or get rid of unwanted thoughts or symptoms of emotional distress; rather, it promotes acceptance of them. For this reason, ACT is part of the newer, “mindfulness and acceptance” wave of behavioral therapies made popular over the past 20 years. As a psychotherapy, ACT is an efficacious treatment for a variety of clinical disorders, including social anxiety (Kocovski et al., 2013; Craske et al., 2014; Herbert et al., 2018), depression (Tamannaeifar et al., 2014; A-Tjak et al., 2018; Pleger et al., 2018), obsessive–compulsive disorder (OCD) (Ghasemi et al., 2017; Rohani et al., 2018; Twohig et al., 2018), chronic pain (Thorsell et al., 2011; Wetherell et al., 2011; McCracken et al., 2013), substance use disorders (Luoma et al., 2012; Lanza et al., 2014; Azkhosh et al., 2016), and others. It is also efficacious when used in non-clinical settings, to help enhance athletic performance (Lutkenhouse et al., unpublished; Gross et al., 2016; Josefsson et al., 2019), to improve workplace performance (Bond and Bunce, 2003; Bond and Flaxman, 2006; Bond et al., 2016), to lessen procrastination in college students (Scent and Boes, 2014; Gagnon et al., 2016; Wang et al., 2017), to serve as a smoking cessation treatment (Hernandez-Lopez et al., 2009; Bricker et al., 2017; O’Connor et al., 2020), and others. When used with non-clinical populations, ACT is often referred to as “acceptance and commitment training” or “acceptance and commitment coaching” (ACC).

The hallmark of the ACT and ACC models is that psychological inflexibility is what leads humans to suffer. By psychological inflexibility, ACT is referring to the combination of several, ongoing *behavioral processes* that are expressed both internally (privately) and externally (publicly) and lead to the development and maintenance of numerous psychological problems. These processes are the following: a lack of awareness and acceptance of present moment experiences; regular avoidance of unwanted internal experiences, such as thoughts, emotions, and sensations (even when doing so creates negative consequences for them); being overly entangled with or fused with one’s thinking; rigid attachment to a conceptualized version of oneself that is insensitive to the contextual influences on their behavior; lack of values or lack of values clarity; and an inability to build larger patterns of behavior through a commitment to actions consistent with one’s values (Hayes et al., 1999, 2011). When any or all of these processes are present, the result is an inflexible pattern of behavior that interferes with growth and correlates with numerous psychological problems.

The goal for an ACT treatment, then, is to enhance psychological flexibility so the person may become less stuck in any of these unhelpful behavioral processes. Improving psychological flexibility requires training in persisting with behavior of personal value to someone, even if doing so includes the presence of unwanted discomfort. The flexibility that comes from tolerating unwanted internal experiences while *simultaneously* engaging in valued actions (and making a commitment to regularly behave this way) correlates with a host of desired outcomes in both clinical and non-clinical settings, like increased well-being (Wersebe et al., 2018), improved ability to interact in social settings (Herbert et al., 2018), improved public speaking ability (Glassman et al., 2016), improved quality of life for depressed patients (A-Tjak et al., 2018), decreased pain interference in daily life (Wetherell et al., 2011), reduced drug use (Lanza et al., 2014), and the performance outcomes already mentioned for athletes and workers (Bond and Bunce, 2003; Bond and Flaxman, 2006; Lutkenhouse et al., unpublished; Bond et al., 2016; Gross et al., 2016; Josefsson et al., 2019). Newer ACT for MPA psychotherapy research also shows that similar, performance enhancement outcomes have occurred with musicians; for example, an undergraduate violinist earned higher performance ratings by a juror after 10 sessions of ACT (Juncos and Markman, 2015), and seven vocal students showed a statistically significant improvement in their performance ratings from two classically trained jurors, after 12 sessions of ACT (Juncos et al., 2017). In addition, there is growing consensus among ACT researchers that psychological flexibility is a trainable set of skills that promote various health outcomes for the general public (Gross et al., 2016; Gloster et al., 2017b).

**FIGURE 1:**
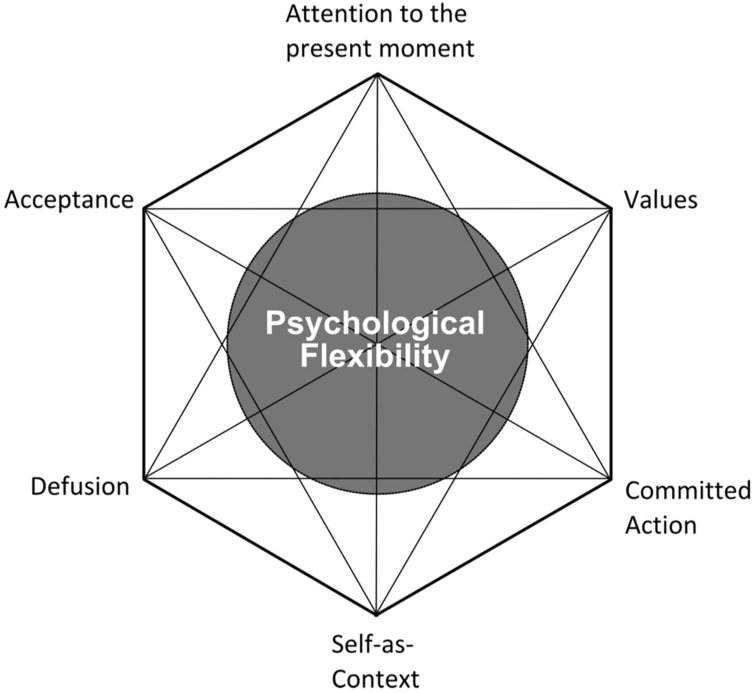
The ACT “Hexaflex.”

As mentioned, the goal for an ACT treatment is not to reduce symptoms of MPA. Ironically, a reduction in MPA symptoms usually does occur, although it is not the direct aim (Bach and Moran, 2008), and it usually occurs when musicians give up the struggle against their MPA symptoms (Juncos and Markman, 2015; Juncos et al., 2017). The six behavioral processes that create psychological flexibility in ACT are known as the “ACT Hexaflex (Hayes et al., 2006),” and they are the following: mindfulness of the present moment, acceptance and willingness toward one’s discomfort, cognitive defusion, cultivation of an observer or transcendental sense of oneself, identification of one’s values, and committed action to one’s values (Figure 1; Batink et al., 2016). The ACT practitioner aims to improve upon each of the Hexaflex processes through discussion, metaphor, meditations, experiential exercises, and out-of-session homework. Targeting specific Hexaflex processes is made easier though the use of self-report measures to identify which processes are in need of improvement.

### Aims of This Study

This study aimed to be the first application of ACC in which a singing teacher administered the interventions directly to a student vocalist with problematic MPA, using a single-subject design. The research question to be answered was this: Can a singing teacher, without education or training in psychotherapy, achieve the same result with a student vocalist with problematic MPA as a clinical psychologist achieved when treating seven student vocalists with problematic MPA over 12 sessions of ACT psychotherapy, when that teacher is trained in ACC by the psychologist? We hypothesized there would be a significant improvement in the current student’s psychological flexibility after receiving six ACC sessions from a singing teacher and that these improvements would be maintained 3 months afterward. Specifically, we predicted that there would be significant improvements in the student’s defusion and acceptance of MPA after the six ACC sessions and that they would be maintained 3 months later. The ability to defuse from MPA-related thoughts (to disentangle oneself from their MPA-related thoughts so that they are less reactive to them during performances) and an increased acceptance of one’s cognitive and physiological MPA symptoms are two proposed mechanisms by which musicians with MPA increase their psychological flexibility (Juncos et al., 2014, 2017; Juncos and Markman, 2015; Juncos and de Paiva e Pona, 2018). No hypothesis was made about whether a reduction in MPA symptoms would occur after the ACC intervention, as ACC does not aim to reduce symptoms. However, a measure of MPA symptoms would still be used to see how ACC would affect the student’s MPA.

## Materials and Methods

### Participant

The participant was a 19-year-old, male, music theatre major and vocalist at the same university where the primary author (TS) worked as a singing teacher, the University of Chichester. He will be referred heretofore as “Toby,” a pseudonym. Toby responded to a mailing for students in Levels 4 and 5 (first and second years of university) asking for participants in a coaching study to help with MPA. He just completed his Level 4 year and reported having problematic levels of MPA, including cognitive symptoms, physiological arousal symptoms, overt avoidance of classroom singing and auditioning for desired roles, and distress over having MPA but no real impairment. He also mentioned to TS he felt very uncomfortable enrolling in psychotherapy to treat his MPA, due to the stigma of being a therapy patient, and instead, he was only comfortable participating in a coaching context.

### Coach

TS is an associate lecturer and singing teacher at Chichester University, while also working in private teaching practice and as an examiner for Trinity College London. She was trained as a classical singer with an international career in opera, concerts, and contemporary classical music. She became intrigued by the occurrence of MPA among her undergraduate students and created a survey to measure the prevalence of their MPA as part of her M.A. thesis in Voice Pedagogy. Her M.A. program also included education in a popular coaching model, GROW (Whitmore, 1992), that has been widely used to enhance performance at the workforce and with athletes. Although she has extensive experience as a singing teacher, she has had no training or education in psychotherapy. TS had some previous contact with Toby. He attended her performance class during his first semester but overtly avoided performing the entire time.

Institutional review board (IRB) approval for this research was given by the University of Wales Trinity Saint David’s (UWTSD) ethics board. TS conducted this research with the Voice Workshop, Ltd., a provider of postgraduate voice pedagogy study that is affiliated with the UWTSD. TS was supervised by the third author (DW) in securing ethics board approval and in successfully navigating the ethical matters in this study, that is, ensuring TS did not function in the role of psychotherapist to the student and ensuring Toby was aware of available psychotherapy resources if needed.

### Procedure

#### Training

TS received training in ACC by the second author (DJ), a clinical and performance psychologist with 15 years’ experience in treating anxiety disorders and specific expertise in using ACT to treat MPA. DJ has conducted two single-subject designs and a pilot study with seven vocal students, in which ACT was investigated as an MPA treatment. He also provides ACT-based training for singing teachers to help them manage students’ MPA. TS’ total ACC training lasted approximately 7 h via Skype. Her training was split into two halves: 4 h of general education about ACC prior to starting with Toby and approximately 3 h of ongoing consultation about how he was uniquely responding to the ACC coaching after it commenced. During the education half, TS was taught about ACC’s overarching goal of psychological flexibility and how each of the six Hexaflex processes contributes to improved psychological flexibility. This half also specified how each process is taught to clients, either musicians or clinical patients, and how to monitor changes in the processes through the use of self-report measures. The second half of training included TS providing regular reports to DJ about Toby’s progress with the ACC and DJ providing ongoing consultation on what modifications were necessary to ensure he continued to benefit. The ACT self-report measures helped inform DJ of the areas Toby was improving with versus those areas he still needed help with.

Materials for TS’ ACC training and Toby’s coaching were taken from two books: *Acceptance and Commitment Coaching* by Hill and Oliver (2019) and *Acceptance and Commitment Training for Musicians*, an unpublished self-help book for musicians, by DJ and de Paiva e Pona. The ACC book by Hill and Oliver includes a six-session model for administering ACC to clients, which provided the outline for the coaching intervention. More specific materials, including in-session exercises, metaphors, and detailed descriptions of Hexaflex processes were taken from DJ and de Paiva e Pona’s unpublished *ACT for Musicians* book.

#### Coaching Intervention

TS initially met with Toby for an introductory session to discuss the ACC intervention and to obtain his consent. Then, they met for six, 60-min sessions between June and September 2019. Four of the sessions were in person, and two were conducted over Skype in order to better accommodate his schedule. The ACC work included interventions aimed to improve all six Hexaflex processes. Some exercises TS used to achieve these aims were the following: a body scan meditation to promote mindfulness, a thought labeling exercise to promote defusion, a meditation to promote acceptance of his MPA-related thoughts and feelings, identification of his values and eliciting a commitment to engage with his values while performing, and an exercise to lessen his attachment to a comedic side of himself he often portrayed when anxious, because it was limiting his artistic growth. These were taken from DJ and de Paiva e Pona’s unpublished book. When clarifying his values, TS restricted the conversations to his performance-related values only, rather than his general life values, to keep the ACC intervention more relevant to his needs as a performer. Examples of his performance-related values were connecting with the audience and expressing himself, both of which were consistent with other vocal students’ performance-related values (Juncos et al., 2017). When discussing Toby’s commitment to engage in valued actions, TS used a helpful paradigm in ACT in which he learned to sort his behavior into two categories: *away moves*, that is, any behavior that is an attempt to avoid internal, unwanted symptoms of MPA, and *toward moves*, that is, any behavior that is an attempt to move toward external sources of reward or value (Hill and Oliver, 2019). Toby was encouraged to become aware of these two styles of his behavior and to make more *toward moves* during his performances, in spite of his MPA.

The coaching intervention included content from the GROW model, which stands for Goal, Reality, Options, and Will (Whitmore, 1992). This coaching model enabled TS to continually monitor Toby’s behavior during the ACC sessions, by establishing where he was going (the Goal), working out where he was currently (the Reality), exploring the various routes available to take him to his destination (the Options), and establishing the commitment (the Will) to make the journey, understanding that obstacles along the way may necessitate changes in direction. TS regularly adhered to the GROW model throughout her sessions with Toby.

Toby completed self-report measures just before the beginning of the ACC intervention (June 2019), at the midpoint (August), at the end (September), and at 3 months after the ACC ended (December). TS also conducted a semi-structured interview with him during the sixth session to inquire about his experience with the ACC intervention.

#### Self-Report Measures

##### ACT-based measures

The Believability in Anxious Feelings and Thoughts (BAFT; Herzberg et al., 2012) was used to measure his level of cognitive defusion. It uses a 7-point Likert scale to measure how much he agreed with each of the 30 statements related to fusion (where 1 = not at all believable and 7 = completely believable). Higher total scores on the BAFT reflect higher levels of fusion with anxious thoughts and feelings, and it shows excellent internal consistency (α = 0.90).

The Acceptance and Action Questionnaire-2 (AAQ-2; Bond et al., 2011) is a seven-item measure that was used to assess his overall psychological flexibility. It asked him to rate his level of agreement with statements about his ability to behave flexibly in the presence of unwanted cognitive and emotional symptoms, using a 7-point Likert scale (where 1 = never true and 7 = always true). Higher total scores indicate higher levels of psychological inflexibility, and it shows good internal consistency (α = 0.84).

The Philadelphia Mindfulness Scale (PHLMS; Cardaciotto et al., 2008) was used to assess his mindfulness. It is a 20-item measure composed of two subscales: the *awareness* subscale, which measured his continuous monitoring of internal and external experiences, and the *acceptance* subscale, which measured his non-judgmental attitude toward his experiences. Both subscales assessed the degree to which he agreed with various statements about his mindfulness in the past week, using a 5-point Likert scale (where 1 = never and 5 = very often). Higher subscale scores indicate higher levels of each construct. Internal consistency for the PHLMS is adequate for both the *awareness* subscale (α = 0.75) and the *acceptance* subscale (α = 0.82).

##### Symptom-based measure

The revised Kenny Music Performance Anxiety Inventory (KMPAI; Kenny, 2009) is a 40-item measure that assessed the degree to which he agreed with statements about anxiety-related discomfort associated with MPA. It uses a 7-point Likert scale (where 0 = strongly disagree and 6 = strongly agree, or vice versa, depending on the statement). Higher total scores indicate greater levels of anxiety and MPA-related distress. The revised KMPAI shows excellent internal consistency (α = 0.94). Its author also suggests that a score of 105 or higher would indicate clinically significant levels of MPA (Ackermann et al., 2014).

## Results

### Change in ACT-Based Measures

**FIGURE 2:**
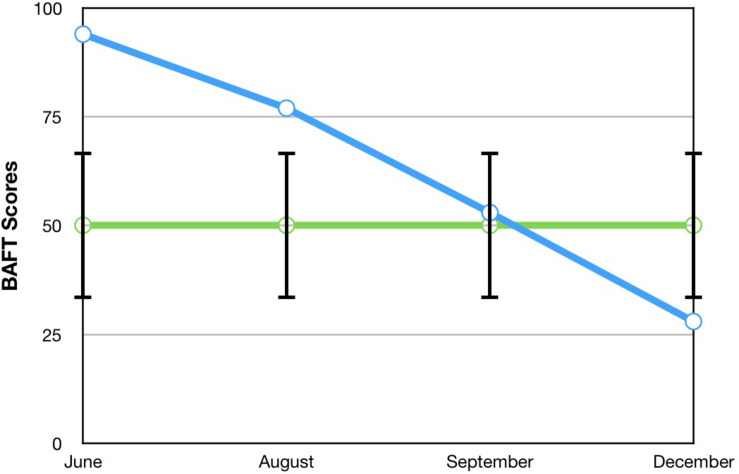
A vocal student’s BAFT scores from June, August, September, and December 2019, with the non-clinical mean (50.10) and bars spanning one standard deviation up/down (1 SD = 16.88; Herzberg et al., 2012).

**FIGURE 3:**
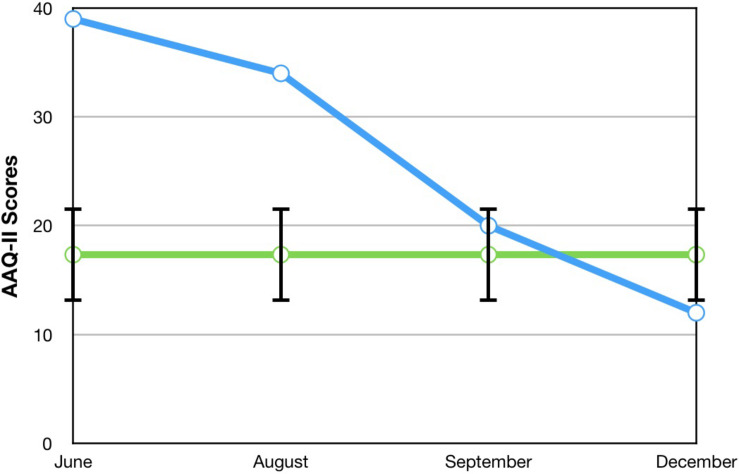
A vocal student’s AAQ-II scores from June, August, September, and December 2019, with the non-clinical mean (17.34) and bars spanning one standard deviation up/down (1 SD = 4.37; Bond et al., 2011).

**FIGURE 4:**
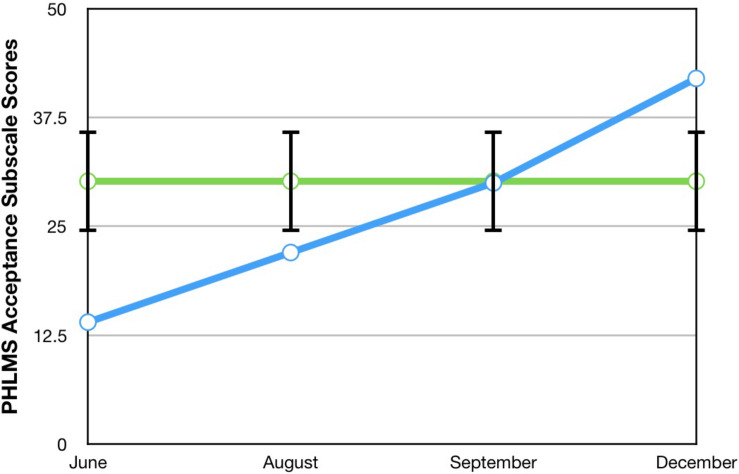
A vocal student’s PHLMS *acceptance* subscale scores from June, August, September, and December 2019, with the non-clinical mean (30.19) and bars spanning one standard deviation up/down (1 SD = 5.84; Cardaciotto et al., 2008).

**FIGURE 5:**
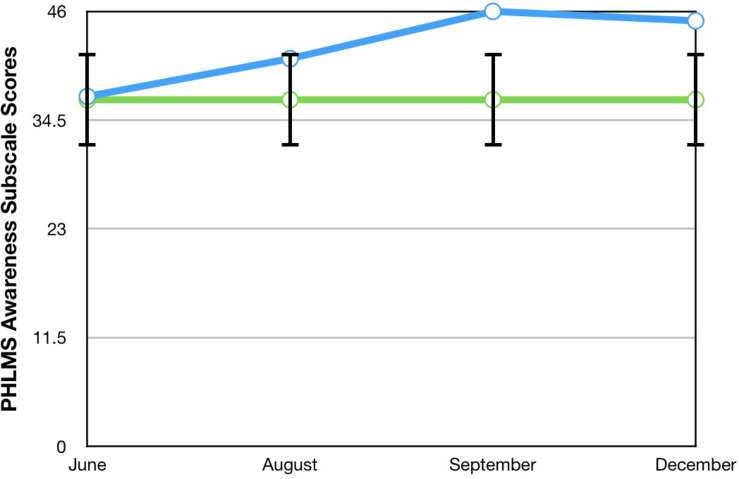
A vocal student’s PHLMS *awareness* subscale scores from June, August, September, and December 2019, with the non-clinical mean (36.65) and bars spanning one standard deviation up/down (1 SD = 4.93; Cardaciotto et al., 2008).

A visual inspection of Toby’s changes on the BAFT, AAQ-II, and both PHLMS subscales (*acceptance* and *awareness*) from June to December 2019 revealed he made improvements on all four measures (see Figures 2–5). Therefore, a reliable change index (RCI; Jacobson and Truax, 1991) was calculated to determine if the observed improvements were reliable or simply due to the standard error of measurement. Each RCI calculation (see Table 1) indicated that a reliable change had indeed occurred at the data collection points in August, September, and December for all four measures, with only one exception: Toby’s scores on the PHLMS *awareness* subscale did not change reliably from June to August. A more stringent test was then applied to evaluate if the reliable changes were clinically significant or not, using Kazdin’s (2011) departure from dysfunction criteria; that is, if a robust change of two standard deviations or more is observed between the pretreatment and post-treatment scores, in the direction of a positive change, the change is considered clinically significant. Table 2 shows that Toby made clinically significant improvements on the BAFT, AAQ-II, and PHLMS *acceptance* subscale at the conclusion of his ACC work in September 2019, and these improvements had progressed further 3 months later, in December 2019. Therefore, the general hypothesis that he would improve his psychological flexibility and specifically that he would improve his ability to defuse from his MPA-related thoughts and become more accepting of his MPA were both supported. He did not make clinically significant improvement on the PHLMS *awareness* subscale during the course of his ACC work, however.

**TABLE 1:** Reliable change indexes (RCIs) for ACT-based measures from June to August, June to September, and June to December 2019 (RCI > 1.96 or < –1.96 = reliable).

Measure	RCI (June–August)	RCI (June–September)	RCI (June–December)
**PHLMS**			
*Awareness*	1.15*	2.58	2.29
*Acceptance*	2.28	4.57	7.99
AAQ-2	−2.02	−7.69	−10.92
BAFT	−2.25	−5.43	−8.74

*^∗^Unreliable change, according to Jacobson and Truax (1991).*

**TABLE 2:** Data for self-report measures from June, August, September, and December 2019, with normative means with standard deviations.

Measure	June	August	September	December	Normative, mean (SD)
**PHLMS**					
*Awareness*	37	41	46	45	36.65 (4.93)^a^
*Acceptance*	14	22	30*	42*	30.19 (5.84)^a^
AAQ-2	39	34	20*	12*	17.34 (4.37)^b^
BAFT	94	77	53*	28*	50.10 (16.88)^c^
KMPAI	−	−	−	69	93.50 (39.10)^d^

^a^Normative means and standard deviations for the PHLMS, ^b^AAQ-2, and ^c^BAFT were taken from non-clinical samples of undergraduates. ^d^Normative mean and standard deviation for the KMPAI was taken from a group of professional orchestral musicians in Australia under age 30. *Clinically significant improvement from June score, as defined by a difference of 2 SD or more from the original score, in the direction of a positive change (Kazdin, 2011).

### Comparisons Between Toby’s Results and Those From Juncos et al. (2017)

**FIGURE 6:**
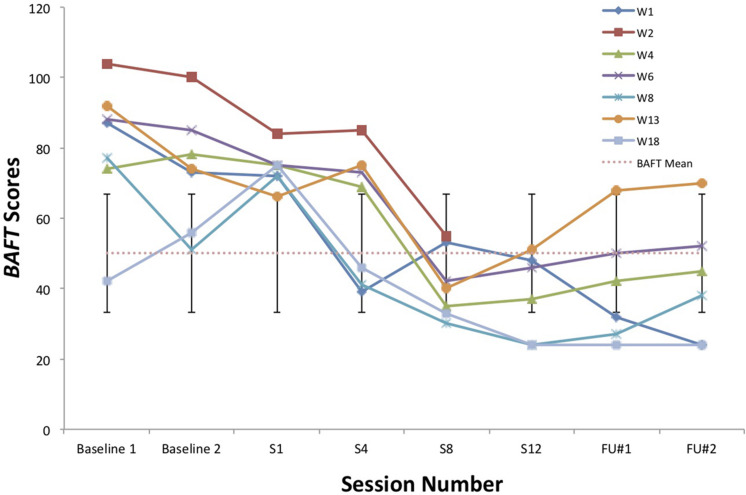
BAFT scores for seven vocal students (Juncos et al., 2017) showing scores from the baseline period to post-treatment, 1- and 3-month follow-up points, with the non-clinical mean (50.10) and bars spanning one standard deviation up/down (1 SD = 16.88; Herzberg et al., 2012).

**FIGURE 7:**
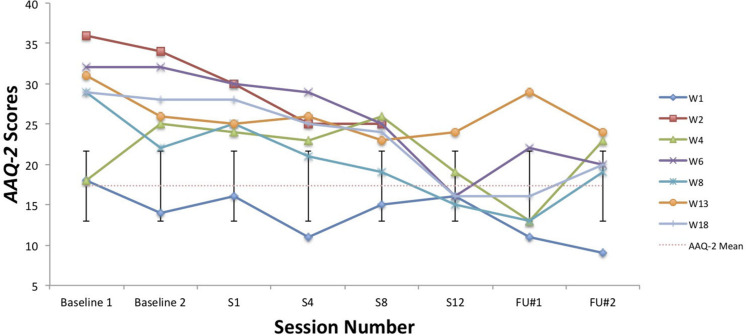
AAQ-II scores for seven vocal students (Juncos et al., 2017) showing scores from the baseline period to post-treatment, 1- and 3-month follow-up points, with the non-clinical mean (17.34) and bars spanning one standard deviation up/down (1 SD = 4.37; Bond et al., 2011).

**FIGURE 8:**
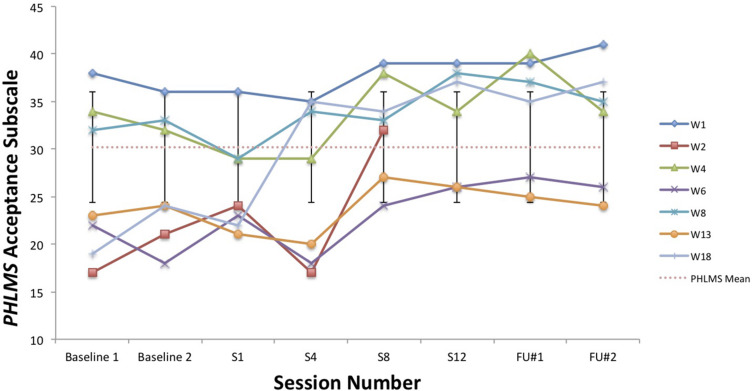
PHLMS *acceptance* subscale scores for seven vocal students (Juncos et al., 2017) showing scores from the baseline period to post-treatment, 1- and 3-month follow-up points, with the non-clinical mean (30.19) and bars spanning one standard deviation up/down (1 SD = 5.84; Cardaciotto et al., 2008).

To compare Toby’s results on his ACT measures with those from the seven vocal students who received 12 sessions of ACT psychotherapy and 1- and 3-month follow-up assessments with a clinical psychologist (Juncos et al., 2017), the reader is guided to Figures 6–8. Figure 6 shows the vocal students’ BAFT data (Juncos et al., 2017) and should be viewed alongside Toby’s BAFT data (Figure 2), Figure 7 shows their AAQ-II data and should be viewed alongside Toby’s AAQ-II data (Figure 3), and Figure 8 shows their PHLMS *acceptance* subscale data and should be viewed alongside Toby’s PHLMS *acceptance* subscale data (Figure 4). The 3-month follow-up point in Juncos and colleagues’ (2017) study is marked as “FU#2” in Figures 6–8.

Toby’s score on the AAQ-II prior to starting his ACC work (39, Figure 3) was worse (higher) than the most impaired vocal student’s score on the AAQ-II prior to beginning the ACT therapy (36, at “Baseline 1” in Figure 7). This indicates that his baseline level of overall psychological flexibility before starting ACC was worse than that of the most impaired vocal student prior to their starting the ACT therapy. However, Toby’s score on the AAQ-II at the 3-month follow-up (12, Figure 3) was similar to the lowest scoring (best scoring) vocal student’s AAQ-II score at the 3-month follow-up in the 2017 study (9, at “FU#2” point in Figure 7). Additionally, Toby’s score on the PHLMS *acceptance* subscale before the start of ACC (14) was also worse (lower) than the most impaired vocal student’s baseline score on the same measure (19, at “Baseline 1” in Figure 8). This indicates his baseline acceptance of MPA was even lower than that of the most unaccepting vocal student in Juncos and colleagues’ (2017) study. However, his score on the PHLMS *acceptance* subscale at the 3-month follow-up (42) was better (higher) than that of the highest scoring vocal student at their 3-month follow-up (41, at “FU#3” in Figure 8).

### Change on Symptom-Based Measure

Unfortunately, Toby was unable to complete the KMPAI at the beginning of his ACC. He did complete it at the 3-month follow-up, however. His follow-up score of 69 (Table 2) is well below the cutoff for determining clinically elevated MPA levels (105). However, since there was no baseline KMPAI score, it was unclear whether he made significant improvement on this measure of MPA. Therefore, it was not possible to determine if his MPA was significantly reduced after the ACC ended.

### Toby’s Impression of Coaching

After coaching ended, Toby reported he found the ACC intervention to be incredibly helpful. Specifically, he found that a “significant shift in his thinking had occurred,” such that he could now accept that “it’s OK to feel anxious, [to] work with it rather than fight it… the idea that it’s OK makes me feel equal to the audience… there’s no judgment.” In addition to becoming more accepting of his MPA, he felt a stronger sense of belongingness to the performing arts community, “I can see that what I am is natural to a performer… it is the way of a performer… I am not alone in this and I can learn to accept it.” He also was excited to start making more *toward moves* in line with his performance-related values. In particular, he volunteered to sing in classes early in the upcoming semester, and he auditioned more for leading roles and eventually won the role of Amos Hart in the musical *Chicago*, both of which were things he previously avoided doing. Lastly, when completing his KMPAI at the 3-month follow-up point, he wrote that he “remains committed to performing and it no longer causes me [him] great anxiety.”

## Discussion

The observed improvements in Toby’s overall psychological flexibility and more specifically the improvements he made in his ability to defuse from MPA-related thoughts and accept his MPA symptoms are consistent with those observed in Juncos and colleagues’ (2017) study, in which seven vocal students who received 12 sessions of ACT psychotherapy for MPA showed a similar pattern of results. It appears Toby specifically learned to defuse from his MPA-related thoughts and to accept his MPA symptoms, and more broadly, he learned to behave more flexibly in the presence of his MPA, equally well as those vocal students who worked with a clinical psychologist. Such a result challenges the long-held notion that music teachers cannot help their students who suffer from problematic MPA in a significant way. When briefly trained in ACC by the second author, it appeared TS was able to identify how Toby was stuck in behavioral processes believed to worsen the struggle associated with problematic MPA, that is, avoidance and fusion, and TS then apparently helped move him through these unhelpful processes, so he became more flexible in the presence of his MPA. As mentioned, TS is a singing teacher without any training or education in psychotherapy. What is also noteworthy is TS was able to achieve clinically significant results with Toby in half the time needed in Juncos and colleagues’ (2017) study, that is, six ACC sessions here compared to 12 ACT psychotherapy sessions. This outcome has important implications for singing (and other music) teachers working with student musicians who suffer from problematic MPA. It suggests a teacher who receives adequate training in an evidence-based coaching model like ACC may be able to help a student with MPA in a significant and relatively quick way, while potentially overcoming some barriers that prevent students from working with a psychologist or other healthcare practitioners, that is, stigma and lack of time/access to services. Furthermore, the specific use of ACC may be more helpful than other coaching approaches, because of its growing research support with non-clinical populations and because it does not require a certification to use (Association for Contextual Behavioral Science, 2020).

Another way TS was able to help Toby was in detecting his MPA was problematic. Unfortunately, he was unable to complete his KMPAI until the 3-month follow-up, so there was no baseline measurement of his MPA. However, it is debatable if a baseline measurement was even necessary. TS was advised by DJ to look for multiple categories of MPA symptoms when recruiting a student for this project, which she was also able to do reasonably well, even without training or education in psychotherapy. Toby showed nearly all categories of MPA symptoms, with the exception of being impaired by it. His baseline scores on the AAQ-II and PHLMS *acceptance* subscale were also worse than any of the vocal students’ baseline scores on the same measures. Therefore, by using this recruitment approach, TS could detect his MPA was indeed problematic and that he needed help, even without the aid of a baseline MPA measurement. Teachers obviously cannot do what psychologists do, in that they lack training and education in the precise evaluation and treatment of mental health disorders through various methods, like the use of validated self-report measures, structured clinical interviews, standardized testing procedures, and the administration of evidence-based psychotherapies. However, they may not require training and education in these methods to help students as long as they are trained in a strategy like the one used here – by looking for multiple categories of symptoms in order to detect a problematic case of MPA. For example, teachers can be trained to recognize when students engage in overt behavioral avoidance or anxious behaviors. The more psychologically attuned teachers may even detect covert behavioral avoidance and private MPA symptoms too, like cognitive symptoms and physiological arousal symptoms.

The results obtained here also raise questions about the need to “outsource” MPA interventions to psychologists or other healthcare practitioners, when music teachers may learn to effectively make the interventions themselves. By referring students with MPA (problematic or not) to be evaluated and treated by a psychologist or another healthcare practitioner, the teacher may remain unaware of the “therapeutic” commonalities between them and psychologists or other practitioners. The results of several psychotherapy meta-analyses have shown a moderate but reliable association between a good therapeutic alliance and a positive psychotherapy outcome, and sometimes this link is more important than the type of psychotherapy used (Horvath and Symonds, 1991; Martin et al., 2000; Shirk and Karver, 2003; Karver et al., 2006; Ardito and Rabellino, 2011). A good therapeutic relationship is commonly thought to include empathy, a congruent or harmonious relationship between client and therapist, and unconditional positive regard (Rogers, 1951), and a good therapeutic alliance is one in which there is agreement on the treatment goals and methods to achieve them, and the development of a personal bond with reciprocal positive feelings occurs (Bordin, 1979). Arguably, these dynamics can extend beyond just the psychotherapeutic relationship, as TS’ relationship with Toby contained elements of a good therapeutic relationship and alliance. Music teachers and coaches who have such relationships and alliances with students are likely to help them.

Furthermore, in outsourcing MPA interventions to psychologists or other healthcare practitioners, music teachers may inadvertently reinforce the idea they cannot be of help. Typically, psychologists and other healthcare practitioners conduct efficacy research for clinical interventions, that is, psychotherapies and medications, and use the results to create best-practice recommendations. Teachers are understandably not involved in this process, given it falls outside of their jurisdiction. However, MPA intervention research should not rely solely on best-practice recommendations borne from efficacy research, which is conducted under highly controlled, “laboratory” settings. What may be more appropriate is to conjointly involve music teachers and psychologists (or other practitioners) in conducting *effectiveness* research for MPA interventions, that is, research that tests how interventions perform under “real-world” conditions, because sometimes the interventions with the most support from efficacy research are not feasible or acceptable in practice (Singal et al., 2014; Orsmond and Cohn, 2015). For example, CBT plus exposure therapy is considered best practice for MPA according to research supporting its efficacy (see Kenny, 2011), but no exposure therapy was used in the current study, and Toby still achieved significant results when working with TS. Toby also made clear it would be unacceptable for him to receive psychotherapy for his MPA. Furthermore, CBT plus exposure must be administered by a clinical psychologist or another qualified mental health practitioner. Considering how numerous music colleges or music departments do not employ psychologists, the real-world conditions in those schools are such that they cannot feasibly follow best-practice guidelines, even though their teaching faculty wants to help. By training music teachers in an evidence-based coaching model like ACC to use with students with MPA, the gap between the laboratory and real-world settings may be narrowed, and best-practice guidelines combining results from *both* efficacy and effectiveness research may be developed with help from music teachers. As a psychotherapy, ACT has already shown efficacy in treating social anxiety disorder (Kocovski et al., 2013; Craske et al., 2014; Herbert et al., 2018), and MPA would be considered a version of that disorder, according to diagnostic criteria in the *DSM-V* (American Psychiatric Association, 2013). Therefore, future research investigating the efficacy of ACT solely as a psychotherapy for MPA may not be needed.

### Limitations and Future Directions

This study is not without limitations. Given it had only one participant and no control condition, direct conclusions about ACC’s effectiveness as an intervention for problematic MPA cannot be made. However, Toby completed the same self-report measures used in a previous ACT for MPA psychotherapy study with vocal students (Juncos et al., 2017), and these measures have been validated on university students. So accurate comparisons could be made between Toby’s scores and those from Juncos and colleagues’ (2017) study, and between his scores and those from the validation samples (Cardaciotto et al., 2008; Bond et al., 2011; Herzberg et al., 2012).

It is possible Toby’s improvements on the ACT-based measures were partly due to a demand characteristic that he was expected to improve, which threatens the internal validity of this study. Evidence in support of this was Toby knew he would be taught again by TS in the upcoming year, and during his coaching, he spoke about the importance of being in good standing with others in his program. However, his pattern of results is similar to those of the vocal students in Juncos and colleagues’ (2017) study and to those of participants in previous ACT for MPA psychotherapy studies (Juncos et al., 2014; Juncos and Markman, 2015). These trends across studies strengthen the possibility that his improvement was actually due to the ACC intervention. Specifically, he engaged in less overt behavioral avoidance after his ACC intervention ended, which was also observed with participants in previous ACT for MPA psychotherapy treatments (Juncos et al., 2014, 2017; Juncos and Markman, 2015). His changing scores on the PHLMS *acceptance* subscale also suggest he struggled less with his MPA symptoms by the sixth ACC session (Table 2). This “lessening of the struggle” with one’s symptoms was observed at approximately the same time in treatment with a student violinist, that is, between sessions 3 and 7 (see Figure 2 in Juncos and Markman, 2015), and with seven vocal students, that is, between sessions 4 and 8 (see Figure 8). In fact, such a result is typically observed *first* in ACT psychotherapy treatments, whereas a reduction in one’s symptoms of distress usually occurs afterward (Bach and Moran, 2008; Juncos and Markman, 2015; Gloster et al., 2017a; Juncos et al., 2017).

A future study should involve more students to see how Toby’s progress might have compared with that of others, using a multiple baseline approach to strengthen its internal validity. Video-recorded performances from before and after the ACC intervention should also be included, so any hypotheses about improvements in observers’ ratings of performance quality may be tested, as in previous ACT for MPA psychotherapy studies (Juncos and Markman, 2015; Juncos et al., 2017). A comparison could also be made in how the ACC intervention is administered: by a teacher working privately with a student(s) or in a classroom setting. Oftentimes, teachers may prefer to administer MPA interventions in the classroom to reach a wider audience, yet others may prefer to devote time in the private lesson to focus more intensely on MPA interventions. Additionally, a future study should compare the GROW model to the ACC intervention to determine either’s strength as an MPA intervention. It is possible TS’ use of the GROW model may have contributed to Toby’s positive results, but the authors were unable to determine the extent of its unique contribution.

It may be beneficial to compare the full, six-session ACC intervention with a shorter one(s) to know the minimum number of ACC sessions needed to produce a clinically significant result on the self-report measures. Significant improvements in Hexaflex processes have been observed when ACT is delivered in brief interventions (some lasting 4, 3 h, or as little as 90 min) and when delivered by therapists without prior ACT experience who received a brief training in it, that is, 15 h. These improvements were also maintained after 1 month, 3 months, 6 months, and 5 years after post-treatment (Eisenbeck et al., 2016; Kohtala et al., 2017; Kroska et al., 2020).

Lastly, we do not wish to promote any oversimplified interpretations of the results. While the obtained results appear positive, we want to discourage the idea that training a music teacher in ACC will lead to developing the same professional competencies as earning a professional certificate or degree in performance coaching would. The latter approach may be more helpful for musicians with MPA than the former. However, the alternative model we proposed in this study appears promising and should be investigated further.

## Conclusion

This single-subject design marked the first investigation into the effectiveness of an evidence-based coaching model, ACC, for use as an MPA intervention with a student vocalist by a singing teacher with no training or education in psychotherapy. The results were promising and suggest ACC may be an effective MPA intervention for use by a singing (or instrumental) teacher with students after a brief training in ACC; however, future research is needed to address this study’s limitations before ACC is considered efficacious and/or effective as an MPA intervention. When used by teachers, ACC may have the potential to overcome some hurdles that often prevent student musicians from seeking help, like the stigma associated with being in therapy and lack of time/access to services. Moreover, this approach may help music schools who do not employ psychologists and are therefore unable to follow current best-practice guidelines for treating MPA. For more information on ACT/ACC, please visit the Association for Contextual Behavioral Science’s webpage (http://www.contextualscience.org).

## Data Availability Statement

All datasets generated for this study are included in the article/supplementary material.

## Ethics Statement

The studies involving human participants were reviewed and approved by the Research Ethics Committee for the University of Wales Trinity Saint David. The patients/participants provided their written informed consent to participate in this study. Written informed consent was obtained from the individual(s) for the publication of any potentially identifiable images or data included in this article.

## Author Contributions

DJ and TS designed the study. TS and DW obtained the IRB approval. DJ provided the ACC training and consultation and supervision to TS on the quantitative data and issues related to clinical significance, and he wrote the manuscript. TS administered the intervention to the student vocalist. DW provided supervision to TS on the study’s ethical matters and on understanding the qualitative data. DJ, TS, and DW edited the manuscript.

## Conflict of Interest

The authors declare that the research was conducted in the absence of any commercial or financial relationships that could be construed as a potential conflict of interest.
